# Role of GATA binding protein 4 (GATA4) in the regulation of tooth development *via* GNAI3

**DOI:** 10.1038/s41598-017-01689-1

**Published:** 2017-05-08

**Authors:** Shuyu Guo, Yuxin Zhang, Tingting Zhou, Dongyue Wang, Yajuan Weng, Lin Wang, Junqing Ma

**Affiliations:** Jiangsu Key Laboratory of Oral Diseases, Nanjing Medical University, Nanjing, China

## Abstract

Transcription factor GATA4 regulates cardiac and osteoblast differentiation. However, its role in tooth development is not clear. Therefore, we generated *Wnt1-Cre;GATA4* ^*fl/fl*^ mice, with conditional inactivation of the GATA4 gene in the dental papilla mesenchymal cells. Phenotypic analysis showed short root deformity along with reduced expressions of odonto/osteogenic markers. Proliferation (but not apoptosis) of cells around the apical area of the root was attenuated. *In vitro*, we knocked down GATA4 expression in stem cells of dental apical papilla (SCAPs). Proliferation, migration and odonto/osteogenic differentiation of SCAPs were affected in the shGATA4 group. Overexpression of GATA4 in SCAPs increased mineralization. Based on our previous iTRAQ results, guanine nucleotide binding proteins 3 (GNAI3) is one of the distinct proteins after GATA4 deletion. G protein signaling is involved in bone development, remodeling, and disease. In this study, both GATA4 deletion in the mouse root and knock-down in human SCAPs decreased the expression of GNAI3. Dual-luciferase and ChIP assay confirmed the direct binding of GATA4 to the GNAI3 promoter, both *in vitro* and *in vivo*. GNAI3 knock-down significantly decreased the odonto/osteogenic differentiation ability of SCAPs. We thus establish the role of GATA4 as a novel regulator of root development and elucidate its downstream molecular events.

## Introduction

Development of tooth root occurs after crown formation and the process involves reciprocal epithelio-mesenchymal interactions^1, 2^. The hard tissue of tooth is comprised of enamel, dentin, and cementum. Dental epithelial cells are the precursors of ameloblasts which in turn form the enamel. Odontoblasts differentiate from dental papilla mesenchymal cells and form the dentin. Dental follicle cells are genuine precursors of cementoblasts which in turn differentiate into cementoblasts. Both odontoblasts and cementoblasts are derived from neural crest cells (NCCs).

GATA binding protein 4 (GATA4) is a zinc finger-containing transcription factor, and is reported to play a vital function in cardiac and intestinal development^3, 4^. GATA4 also regulates the expression of the angiogenic marker vascular endothelial growth factor (VEGF), and VEGF was reported to promote embryonic jaw extension^5, 6^. According to our own previous microarray results (unpublished data), GATA4 expression in the embryonic mouse maxillofacial tissues at embryo day 13.5 (E13.5) was significantly higher than that at E18.5. This result suggests a vital role of GATA4 in the development of the maxillofacial tissues. Besides, several studies have indicated a role of GATA4 in the regulation of osteoblasts^7, 8^. The development of both bone and tooth has several common features. Both MSCs from bone marrows and SCAPs were as potent in osteo/dentinogenic differentiation^9^. This aroused our interest in studying the effect of GATA4 in tooth development. So far, the involvement of GATA4 in tooth development has never been reported. One of the reasons is that global knockout of GATA4 in mice induces severe defects, which results in death of GATA4^−/−^ embryos between 8.5 and 10.5 days post coitum^10^. GATA4^−/−^ embryonic lethality has impeded *in vivo* research into tooth development. Currently, *Wnt1-Cre;GATA4* ^*fl/fl*^ mouse model with conditional knockout of GATA4 in the NCCs-derived dental papilla mesenchymal cells has been developed in this study. This mouse model has facilitated investigation of the role of GATA4 in the regulation of tooth root development. The findings provide insights into the role of GATA4 in tooth morphogenesis and short root deformity, which may facilitate development of new therapies for developmental deformities of the root.

GNAI3 is one of the members of the G protein family, which is also involved in disease of neural crest-derived first and second pharyngeal arch (PA) derivatives^11, 12^. However, the mechanism by which GNAI3 influences tooth morphogenesis, and the relationship between GATA4 and GNAI3 still remains unknown.

Our work indicates a novel role of GATA4 in regulating the proliferation and differentiation of dental mesenchymal cells during tooth development, and that this effect proceeds *via* GNAI3 expression. Moreover, our work showed that GNAI3 mediates these processes under the control of GATA4.

## Results

### Dental papilla mesenchymal cells-specific inactivation of GATA4 leads to root deformity

**Figure 1:**
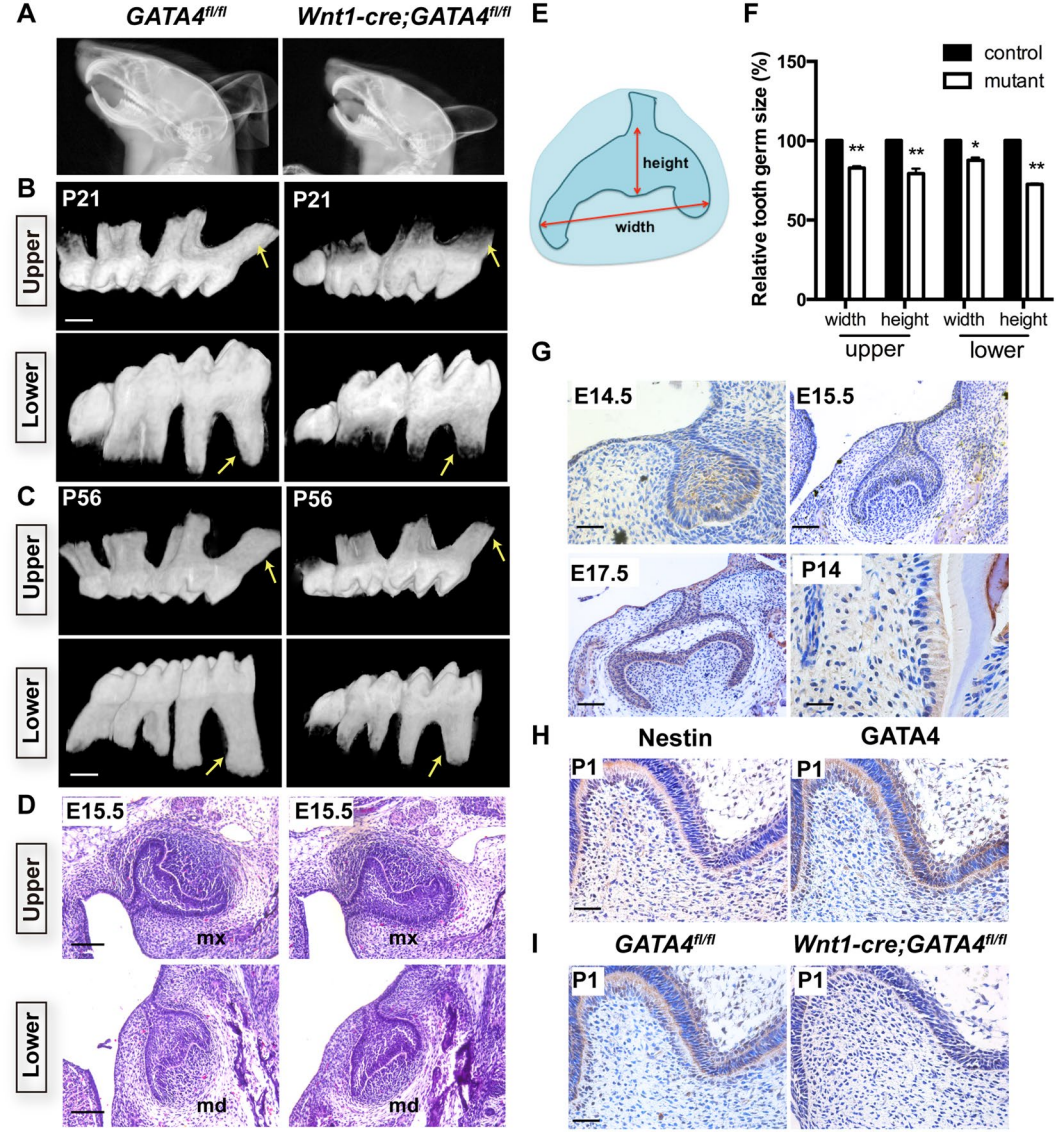
Tooth abnormalities in *Wnt1-Cre;GATA4* ^*fl/fl*^ mice. (**A**) X-ray analysis of the skull; (**B**,**C**) Both control and mutants littermates mice were harvested at P21 and P56 days; Micro-CT showing short roots in the upper and mandibular molars of *Wnt1-Cre;GATA4* ^*fl/fl*^ mice (Bar: 500 μm). (**D**) HE-stained sections showing the development status of upper first molar germ and mandibular first molar germ at E15.5 (Bar: 100 μm); (**E**,**F**) Tooth germ width and height were measured. Quantitative assessment of the molar size in upper first molar germ and mandibular first molar germ; (**G**) Expression pattern of GATA4 during tooth development at embryonic days 14.5 (E14.5, Bar, 50 μm), E15.5, E17.5 and at postnatal day 14 (P14) (Bar: 100 μm); (**H**) Immunostaining for Nestin and GATA4 in P1 (Bar: 50 μm); (**I**) GATA4 expression in tooth of *GATA4* ^*fl/fl*^ and *Wnt1-Cre;GATA4* ^*fl/fl*^ mice at P1 (Bar: 50 μm). Immunostaining confirmed the knockout efficiency of GATA4 in the conditional knockout mice; mx, maxillary; md, mandible.

We removed GATA4 specifically in dental papilla mesenchymal cells by crossing a floxed GATA4 allele with a Wnt1-Cre driver. Unlike GATA4-null mouse embryos which die prior to embryonic day (E) 8.5 to 9.5^10^, *Wnt1-Cre;GATA4* ^*fl/fl*^ mutants were born in the predicted Mendelian ratio and can survive till adulthood. In Fig. 1A, X-ray showed the smaller head of the mutant mice. We used this mouse model to examine the effect of GATA4 on tooth root development. Three–week-old mutants showed short root deformity in both maxillary and mandibular molars (Fig. 1B). By day-56, tooth development is complete. The root of mutant mice at day 56 was found to be longer than that at day 21. However, the tooth root was still shorter than that in the WT mice (Fig. 1C). Compared with control *GATA4* ^*fl/fl*^ littermates, E15.5 mutants exhibited smaller first molar tooth germs, both in the maxillary and mandibular regions (Fig. 1D,E,F). Next, we examined the expression patterns of GATA4 in the mouse mandibular molar tooth germ on immunohistochemical examination at E14.5, E15.5, E17.5, P1 and P14 (Fig. 1G,H). GATA4 protein expression was found in the majority of cells in the dental mesenchyme, odontoblasts and ameloblasts. Odontoblasts were confirmed by the positive expression of Nestin^13^ (Fig. 1H) observed in fully differentiated odontoblasts in the vicinity of dentin in P1 control mice. We also confirmed the knockout efficiency of GATA4 in the conditional knockout mouse. In the *GATA4* ^*fl/fl*^ group, GATA4 was expressed in odontoblasts at P1. However, the expression of GATA4 was not found in the dental mesenchyme and odontoblasts of *Wnt1-Cre*; *GATA4* ^*f*l/*fl*^ mice (Fig. 1I), which confirmed GATA4 deletion in dental papilla mesenchymal cells. In summary, these results indicate that GATA4 plays a role throughout the development of tooth.

### GATA4 deficiency disrupts the differentiation of root dental mesenchymal cells during root formation

**Figure 2:**
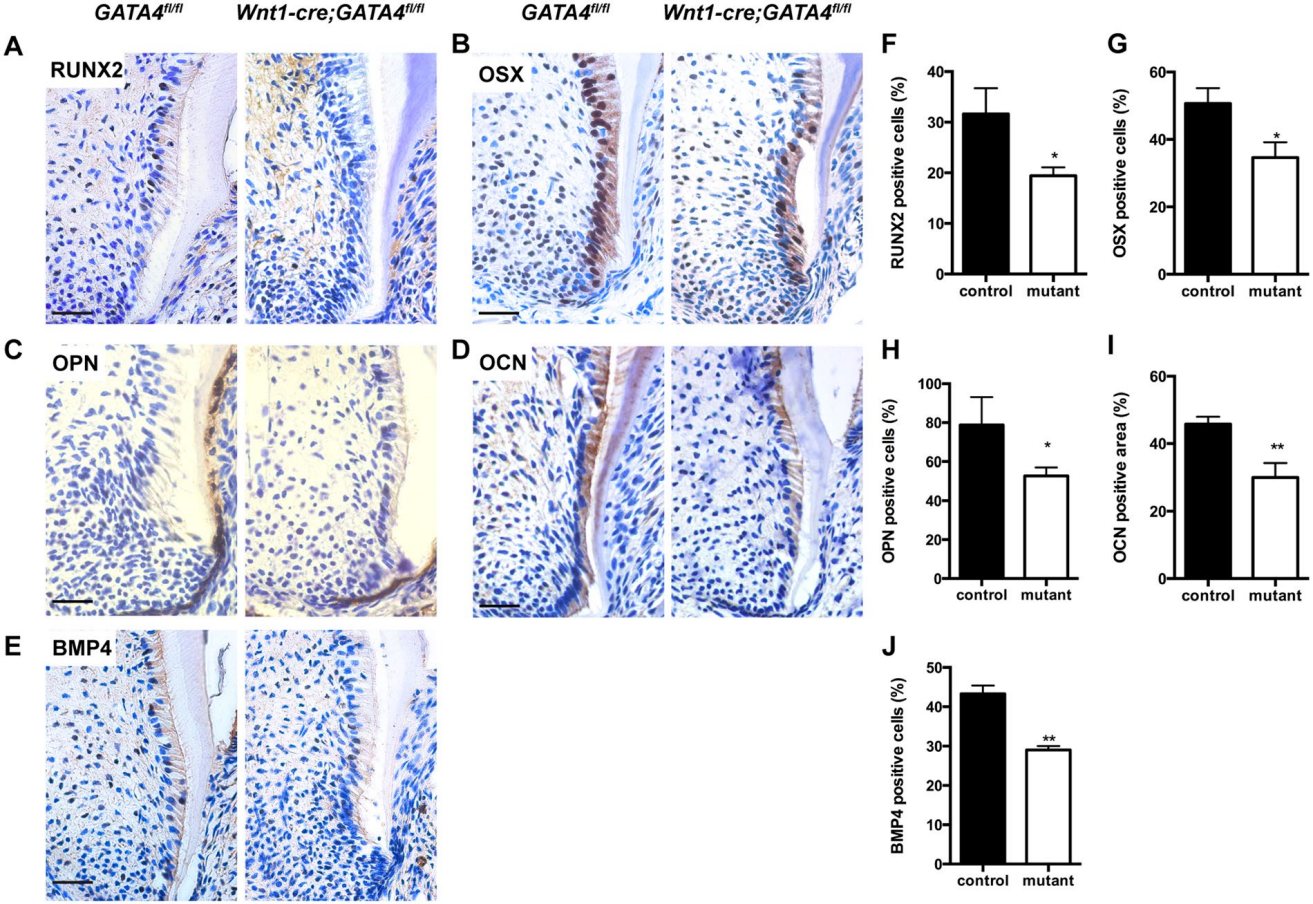
Osteo/odontogenic related changes in gene expression in dental cells. (**A–E**) Immunohistochemistry staining images showing expression levels of RUNX2 (**A**), OSX (**B**), OPN (**C**), OCN (**D**), and BMP4 (**E**) in root of *Wnt1-Cre;GATA4* ^*fl/fl*^ mice at P14. (**F–J**) Percentages of RUNX2 (**F**), OSX (**G**), OPN-positive cells (**H**), OCN-positive area (**I**) and BMP4-positive cells (**J**) in the control and mutant groups were calculated (Bar: 50 μm). *P < 0.05, **P < 0.01.

Molecular changes induced by GATA4 disruption in the mesenchyme of the developing roots of the mandibular first molar were examined by immunohistochemical staining. A reduced expression of RUNX2 was observed in the mutant mouse around the root (Fig. 2A,F). Osterix (OSX) is involved in both root odontoblast differentiation and root formation^14^. In the control mice, OSX-labeled cells were mainly located in dental mesenchymal cells at the apical part of the root. However, the labeled cells located around the root were significantly reduced in the mutant mice (Fig. 2B,G). Further, osteopontin (OPN) and osteocalcin (OCN) were found to be dramatically reduced around the root (Fig. 2C,D,H,I). Bone morphogenetic protein 4 (BMP4) is important for both osteoblast differentiation and tooth morphogenesis^15, 16^. BMP4-labeled cells located around the root were found to be significantly reduced in the mutant mice (Fig. 2E,J).

### Absence of GATA4 in dental papilla mesenchymal cells retards the proliferation but has no effect on apoptosis

**Figure 3:**
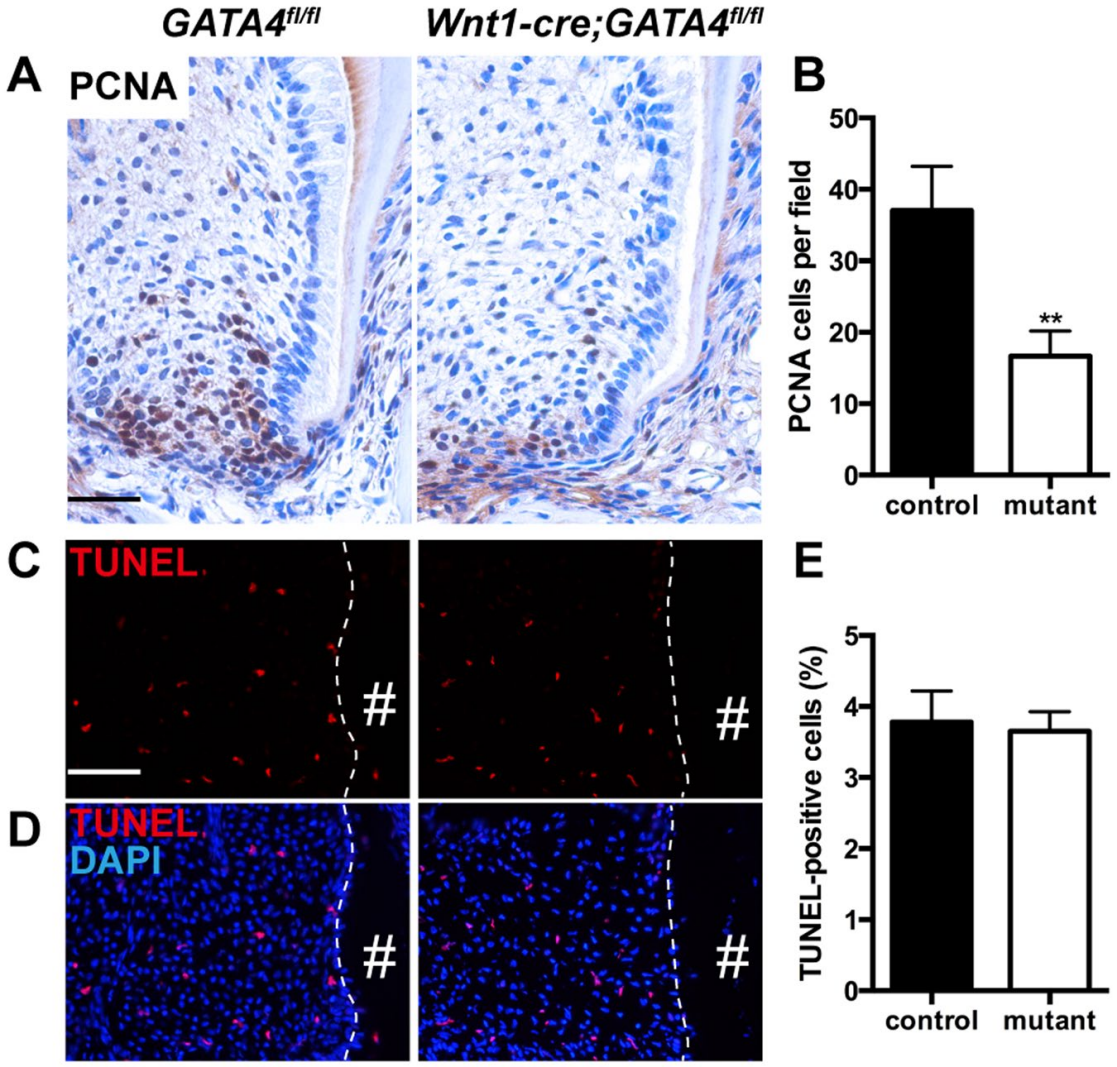
Changes in cell proliferation and apoptosis in the molar dental mesenchymal cells. (**A**) PCNA immunohistochemistry staining; (**B**) Percentages of PCNA-positive cells in the control and mutant groups; (**C**,**D**) Apoptotic cells among the mice root dental mesenchymal cells were identified by TUNEL analysis. Sections were immunostained with markers of cell death, TUNEL (red) and counterstained with DAPI (blue); (**E**) Percentages of TUNEL positive cells in control and mutant are presented (Bar: 50 μm). ^#^Indicates the root of mandibular molars. **P < 0.01.

The findings described here suggest that ablation of GATA4 in the dental papilla mesenchymal cells resulted in the truncation of tooth root by disturbing the molecular expression. We therefore examined whether reduced root growth in the *Wnt1-Cre;GATA4* ^*fl/fl*^ mutants is proceeded by defective cell proliferation or apoptosis in the apical part of the first molar. We used proliferating cell nuclear antigen (PCNA) immunohistochemistry and terminal deoxynucleotidyl transferase dUTP nick end labeling (TUNEL) assay to label the proliferating or apoptotic cells. In the *GATA4* ^*fl/fl*^ mice first molar at P14, proliferating cells were condensed in the radicular portion of tooth roots, which indicated increased proliferative ability of root dental mesenchymal cells along with tooth root development. However, in the *Wnt1-Cre;GATA4* ^*fl/fl*^ mutants, PCNA-labeled cells in the root dental mesenchyme were decreased (Fig. 3A,B). We also assessed the proliferation of HERS, and no significant difference was observed in this respect between the WT and mutant mice (data was not shown). Nonetheless, no significant between-group difference was observed with respect to TUNEL-positive cells around the root dental mesenchyme (Fig. 3C,D,E).

### Characterization of isolated SCAPs and efficiency of GATA4 knock-down

**Figure 4:**
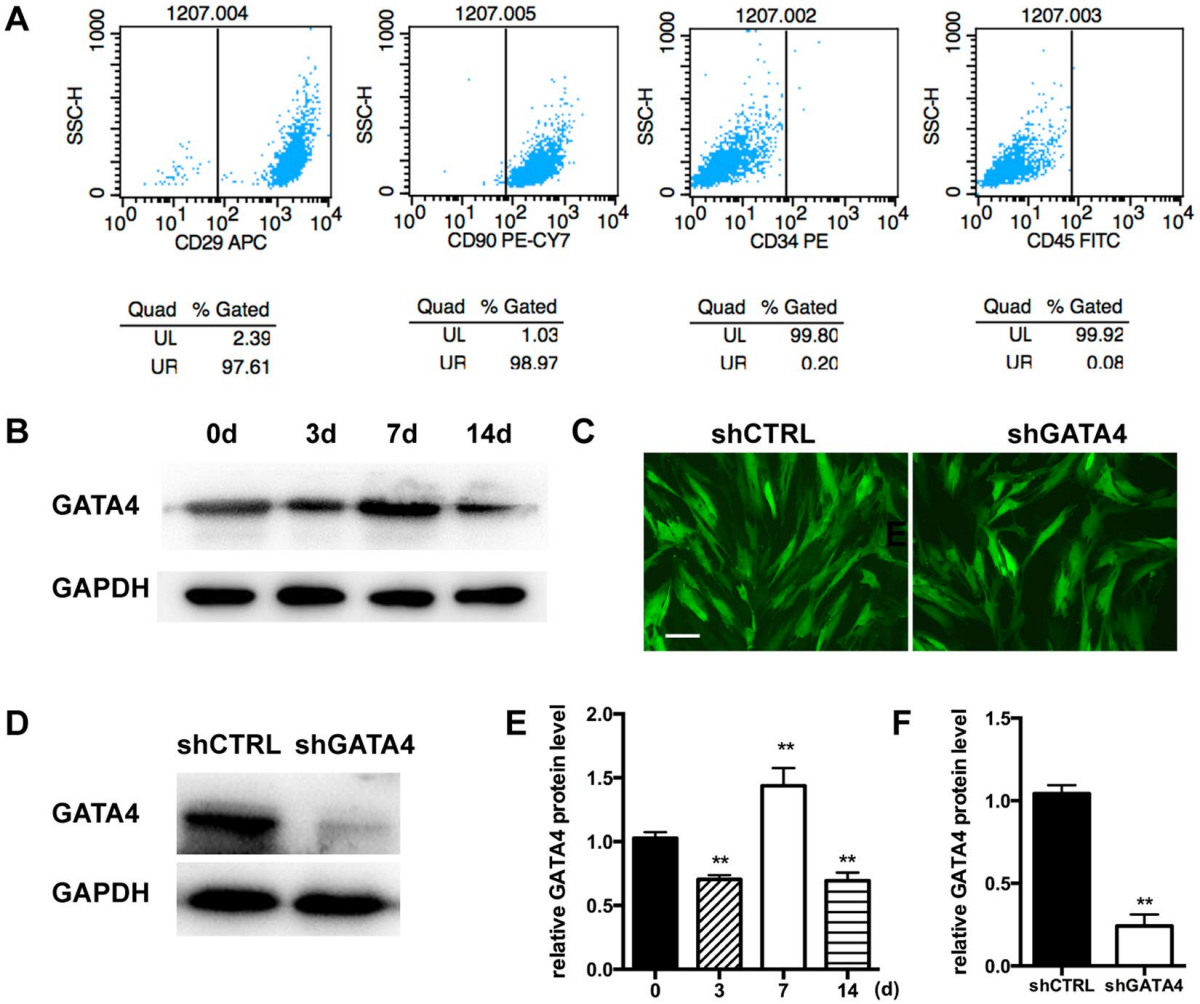
Characterization of SCAPs and expression of GATA4 in SCAPs. (**A**) FCM analysis of SCAP surface markers (CD29, CD90, CD34, CD45); (**B**) After mineralization for 3, 7, and 14 days, the expression of GATA4 protein was assessed at the indicated time points by Western blotting; (**C**) SCAPs infected with lentivirus under fluorescence microscope; (**D**) Efficiency of GATA4 knock-down after infection with lentivirus was analyzed by Western blotting; (**E**,**F**) Quantitative analysis of Western blotting bands from (**B**,**D**) are shown as the ratio of GATA4 to GAPDH (Bar: 50 μm). Data expressed as mean ± Standard Deviation, n = 3. **P < 0.01.

To investigate the effect of GATA4 on SCAPs *in vitro* assays, experiments were performed as below. Flow cytometric analysis revealed positive expression of mesenchymal stem cell (MSC) markers CD29 and CD90 in the *ex vivo*-expanded cells. The hematopoietic markers CD34 and CD45 were almost undetected in SCAPs (Fig. 4A). We checked the expression of GATA4 in SCAPs during osteogenic differentiation. Western blotting showed that GATA4 expression increased during the first seven days of osteoblast differentiation; however, the GATA4 expression at day-14 was lower than that at day-7 (Fig. 4B,E). To assess the function of GATA4 in SCAPs, we used lentivirus-mediated infection with a specific shRNA to knock down GATA4 expression. When SCAPs were transfected by lentiviral particles (MOI 50), we observed green fluorescence in both shCTRL and shGATA4 groups at 72 hours (Fig. 4C). Western blotting analysis showed that the expression of GATA4 in the shGATA4 group was significantly lower than that in the shCTRL group (Fig. 4D,F).

### Effect of GATA4 on migration, proliferation and Odonto/Osteogenic differentiation of SCAPs

**Figure 5:**
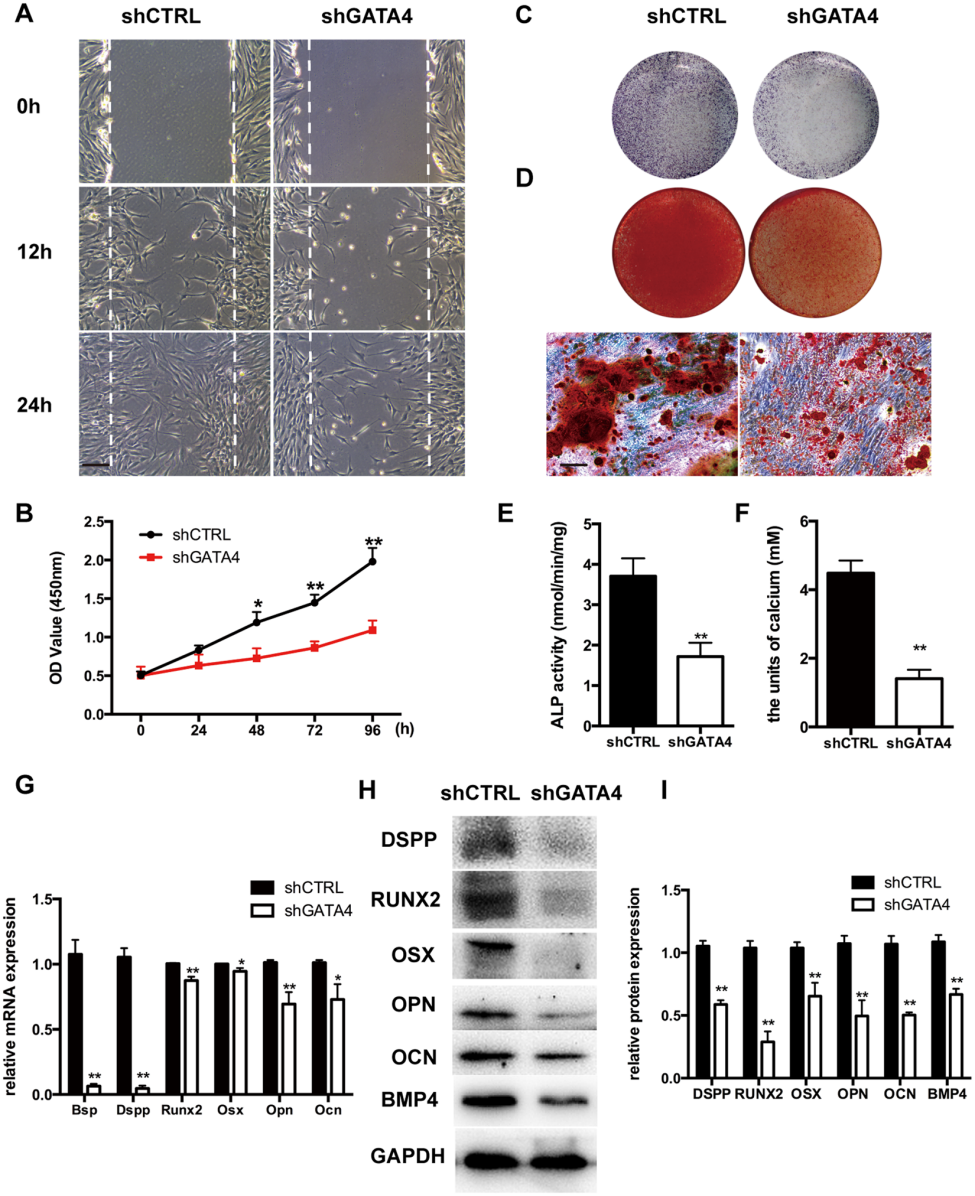
Effect of GATA4 on migration, proliferation and odonto/osteogenic differentiation of SCAPs. (**A**) Effect of GATA4 knockdown on cell migration was assessed by wound scratch; (**B**) CCK8 assay was used to analyze the proliferation of SCAPs after infection with GATA4 lentivirus; (**C**) ALP staining observed after 5 days of mineralization; (**D**) After mineralization for 14 days, Alizarin Red staining was conducted and observed with an image scanner (upper) and under microscope (lower); (**E**) Quantitative assessment of ALP activity; (**F**) Semi-quantitative estimation of calcium; (**G**) Expression levels of osteo/odontogenic related genes (Bsp, Dspp, Runx2, Osx, Opn, and Ocn) were assessed by qRT-PCR; (**H**) Expressions of osteo/odontogenic markers (DSPP, RUNX2, OSX, OPN, OCN and BMP4) were assessed by Western blotting; (**I**) Quantitative analysis of western blotting bands from (**H**) (Bar: 100 μm). Data expressed as mean ± Standard Deviation; n = 3. *P < 0.05, **P < 0.01.

**Figure 6:**
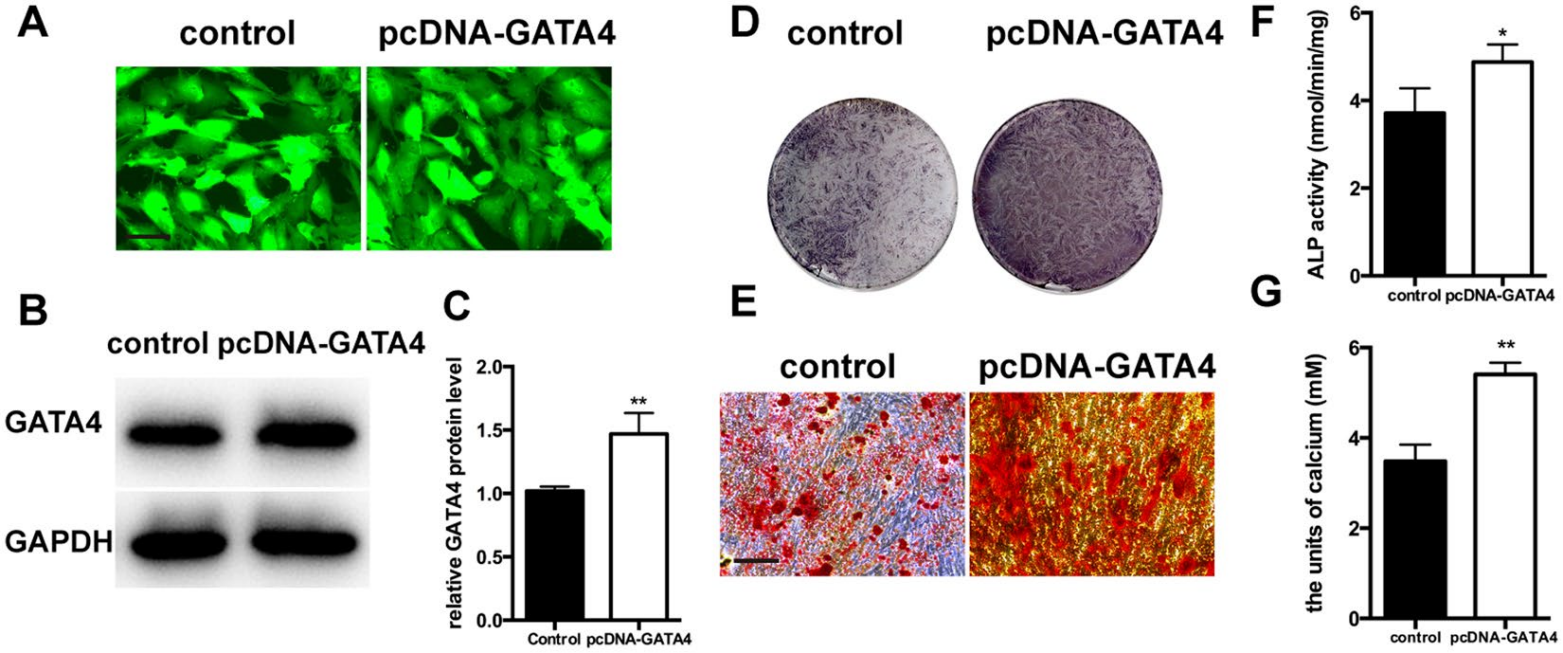
Overexpression of GATA4 in SCAPs increased the osteo/odontogenic ability. (**A**) SCAPs infected with lentivirus control and pcDNA-GATA4 were observed under fluorescence microscope (Bar: 50 μm); (**B**) Protein expression of GATA4 in the SCAPs was tested by Western blotting; (**C**) After GATA4 overexpression in SCAPs, the efficiency of GATA4 knock-down post-lentiviral infection was assessed by Western blotting; (**D**) ALP staining was observed after 5 days mineralization; (**E**) ARS staining was performed 14 days after mineralization (Bar: 100 μm); (**F**) Quantitative assessment of ALP activity after 5 days of osteogenic induction; (**G**) Semi-quantitative estimation of calcium. Data expressed as mean ± Standard Deviation; n = 3. *P < 0.05, **P < 0.01.

As shown in Fig. 5, SCAPs in the shGATA4 group exhibited attenuated migratory ability as shown by the slower scratch closure (Fig. 5A). CCK-8 assays revealed that GATA4 knock-down decreased the proliferative ability compared with control groups after 1, 2, 3, and 4 days of culture, which indicates a significant effect of SCAPs on proliferation (Fig. 5B). For examining the odonto/osteogenic differentiation ability of GATA4, we treated the SCAPs with lentiviral particles for 24 hours, and then changed with the mineralization-induced media. ALP activity was detected by ALP kit after treating the cells with mineralization-induced media for 5 days. shGATA4 group showed lower ALP-positive area as compared to that in the shCTRL group (Fig. 5C,E). Moreover, ARS staining performed 14 days after osteogenic induction, showed markedly decreased the density of calcium nodes in the shGATA4 group (Fig. 5D,F). To prove the results further, we examined the expression of specific osteo/odontogenic genes. Differential expression levels were investigated on qRT-PCR assays. The expressions of Bsp, Dspp, Runx2, Osx, Opn, Ocn were decreased in the shGATA4 group (Fig. 5G). Western blotting assay also revealed a significant decrease in the expression of osteo/odontogenic markers (DSPP, RUNX2, OSX, OPN, OCN, BMP4) in the shGATA4 group (Fig. 5H,I). Then we confirmed the GATA4 overexpression efficiency (Fig. 6A,B,C). Conversely, in the pcDNA-GATA4 group with over-expression of GATA4 in SCAPs, the ALP activity and calcium nodes were markedly increased as observed on ALP assay and ARS staining (Fig. 6D,E,F,G).

### GATA4 knock-down affects GNAI3 expression

**Figure 7:**
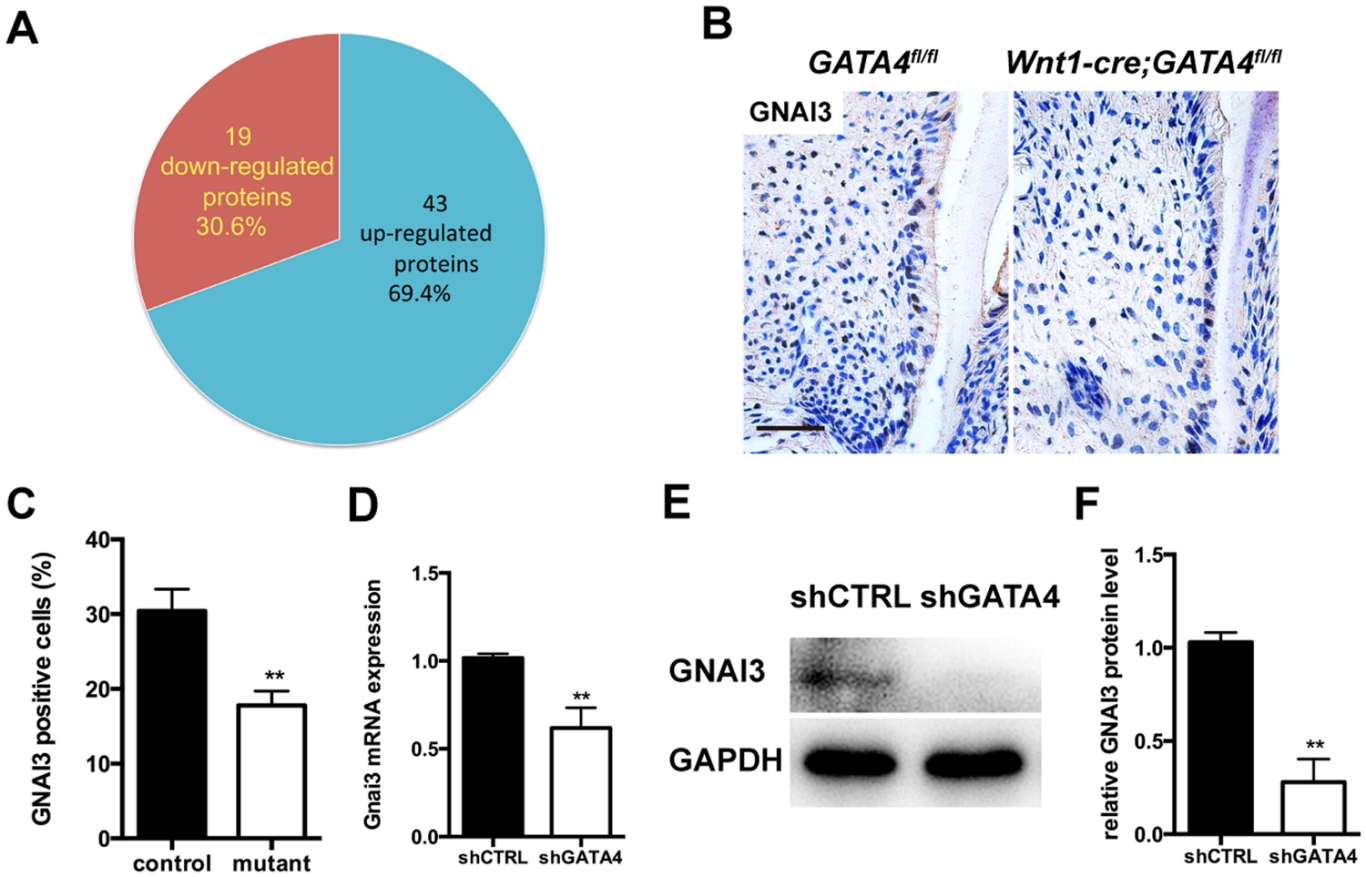
GATA4 knockdown in neural crest cells affects GNAI3 expression both *in vivo* and *in vitro*. (**A**) Forty-three proteins were up regulated and 19 proteins down regulated in the GATA4 knockdown group of neural crest cells, as assessed by iTRAQ assay. GNAI3 was down regulated in the GATA4 knockdown group; (**B**) Immunohistochemistry staining images showing expression levels of GNAI3 in root dental mesenchymal cells (Bar: 50 μm); (**C**) Percentages of GNAI3 positive cells in the control and mutant groups; (**D**,**E**,**F**) mRNA and protein expression levels of GNAI3 in SCAPs was tested by qRT-PCR and western blotting after GATA4 knock-down. Data expressed as mean ± Standard Deviation, n = 3 **P < 0.01.

In our earlier unpublished study, iTRAQ analysis of NCCs which are progenitor cells of SCAPs after GATA4 knock-down revealed a downregulation of GNAI3 in the shGATA4 group (Fig. 7A). In the present study, we assessed the expression of GNAI3 both *in vivo* and *in vitro*. Decreased expression of GNAI3 was found in the root dental mesenchymal cells in the GATA4 group (Fig. 7B,C). Subsequent to this, we induced GATA4 knockdown using lentivirus-mediated infection with a specific shRNA. We assessed the mRNA and protein expressions of GNAI3 in the SCAPs. The expression level of GNAI3 was found markedly decreased in the shGATA4 group. These observations were confirmed by quantitative analysis (Fig. 7D,E,F).

### GATA4 binds to GNAI3 promoter and up-regulates GNAI3 in SCAPs

**Figure 8:**
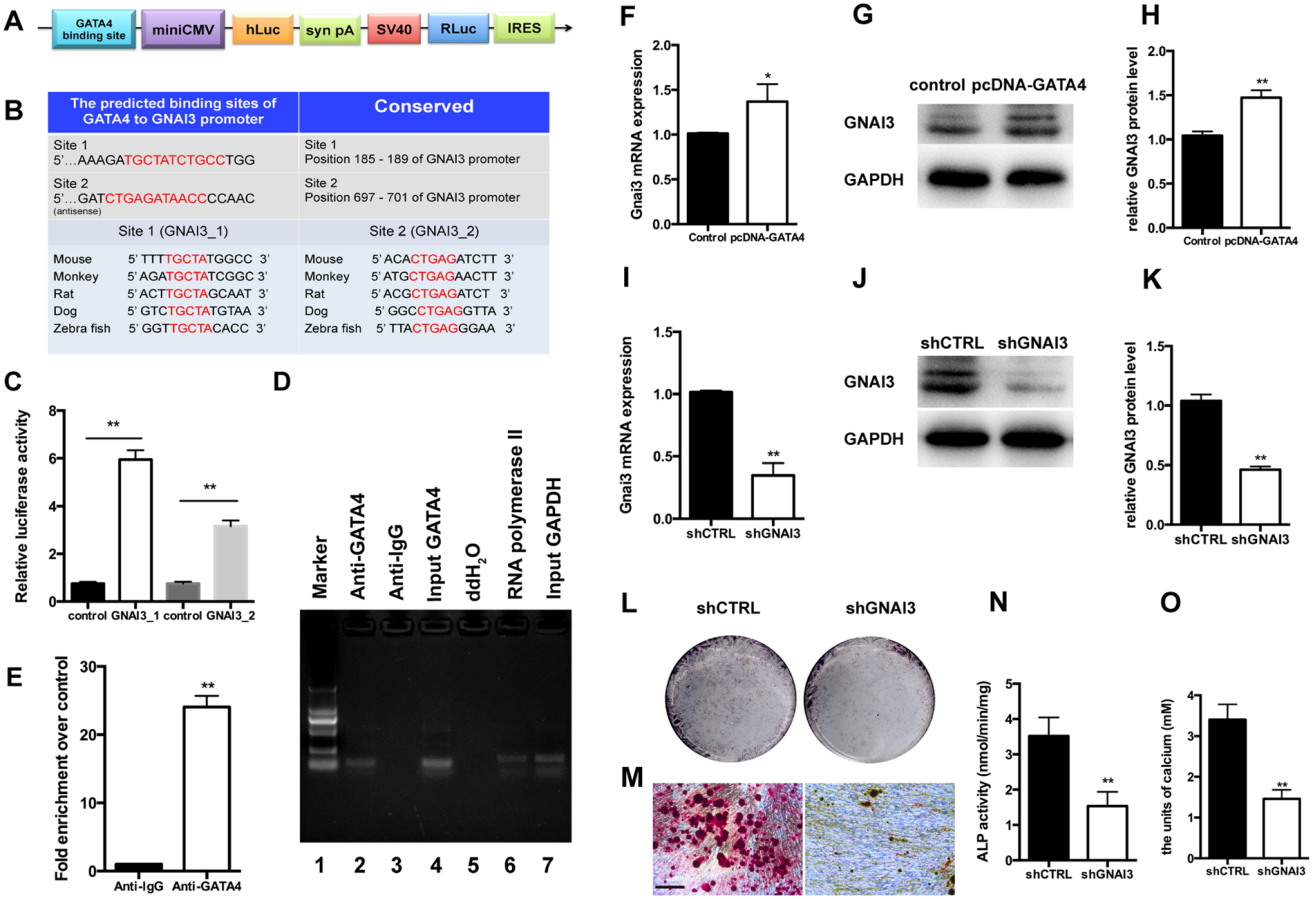
Regulation of GNAI3 expression by GATA4. (**A**) Schematic diagram of the Dual-Luciferase reporter vector (pEZX-FR03); (**B**) By computational target prediction analysis, we selected two binding sites for further research. The matched sequence of GATA4 to GNAI3 promoter was acquired from the JASPAR database. The binding site sequence that is highly conserved (red fonts) in several species; (**C**) Quantitative results of dual-luciferase reporter assay. (**D**) ChIP gel shift assay and quantitative PCR for GATA4 binding to GNAI3 promoter in SCAPs. Lane 1, DNA marker; Lane 2, ChIP sample with GATA4 antibody; Lane 3, ChIP sample with lgG antibody; Lane 4 Input amplified by GATA4 primers; Lane 5, ddH2O amplified by GATA4 primers; Lane 6, ChIP sample with RNA polymerase II antibody; Lane 7, Input amplified by GAPDH primers. (**E**) Primers were designed to cover the GNAI3 promoter region and used to identify the binding sites of GATA4 by ChIP assay. Relative enrichment of the binding sites of GATA4 was observed in SCAPs on qRT-PCR. qRT-PCR (**F**) assay and western blotting (**G**,**H**) were conducted to examine the expression of GNAI3 after GATA4 over-expression. The efficiency of GNAI3 knockdown was analyzed by qRT-PCR (**I**) and western blotting (**J**,**K**). ALP staining (**L**) was examined and activity of ALP (**N**) was analyzed by ALP activity assay kit after 5-days of osteogenic induction; (**M**,**O**) ARS staining was performed and quantified using a microplate reader at 562 nm (Bar: 100 μm). Data expressed as mean ± Standard Deviation, n = 3. *P < 0.05; **P < 0.01.

To investigate the mechanism of cellular regulation in SCAPs, we predicted the interaction between GATA4 and GNAI3 in the database. In the promoter area of GNAI3, there were several binding sites for transcription factor GATA4. This implies that GATA4 may regulate the expression of GNAI3 gene by mediating the transcription of GNAI3. We carried out dual-luciferase assay to verify if GATA4 affected the expression of GNAI3, and we chose two predicted binding sites (GNAI3_1 and GNAI3_2) with high scores for additional research (Fig. 8A,B). Overexpression of GATA4 significantly promoted GNAI3 promoter reporter activity, and the first binding site was found to be better than the second one (Fig. 8C). The first binding site was chosen for further research. To address this, we performed the chromatin immunoprecipitation (ChIP) assays with specific antibodies against human GATA4 in SCAPs. Significant enrichment of GATA4 was identified in the first binding sites in the human GNAI3 promoter region (Fig. 8D,E). qRT-PCR and Western blotting assay also confirmed that overexpression of GATA4 induced an increase in GNAI3 expression both at the mRNA and protein levels (Fig. 8F,G,H). To prove the hypothesis that GATA4 affected odonto/osteogenic differentiation of SCAPs by regulating GNAI3 function, we analyzed the role of GNAI3 in osteoclast differentiation by inducing GNAI3 knock-down. Firstly, we confirmed the efficiency of knockdown GNAI3 by qRT-PCR and western blotting analysis (Fig. 8I,J,K). Subsequently, we performed ALP and ARS staining assays after culture of SCAPs with mineralization-induced media. In the shGNAI3 group, the ALP-positive area and the density of calcium nodes decreased significantly (Fig. 8L,M,N,O). These results suggest that GATA4 modulates odonto/osteogenic differentiation of SCAPs and that this effect is mediated *via* up-regulation of GNAI3 function.

## Discussion

Root hypoplasia is related to the pathogenesis of several craniofacial and oral disorders. Understanding the mechanisms involved in regulation of root growth will facilitate innovative means to diagnose and treat these disorders. In this study, we demonstrated that GATA4 is essential for root formation as it promotes the proliferation of dental mesenchymal cell around the root and affects odonto/osteogenic differentiation of SCAPs *via* up-regulation of GNAI3.

GATA4 is well known for its central role in cardiac development^4^ and osteoblastic differentiation. Gustavo *et al*.^8^ reported that GATA4 is a possible pioneer factor in osteoblasts, which regulates osteoblast-specific genes by recruiting ERα to DNA in osteoblasts. GATA4 is also essential for estrogen-mediated osteoblast gene regulation *via* the ERα and TGFβ/BMP pathways^7^. However, the role of GATA4 in tooth development is not well-characterized as GATA4-null mouse dies before tooth root formation. The tooth phenotype, therefore, is not known, and further exploration of GATA4 function in root is warranted. In our study, the conditional knockout mouse *Wnt1-Cre;GATA4* ^*fl/fl*^ exhibited the short root deformity, which can be used as a novel animal model to study root development.

Currently, the effect of transcription factors on dental root-forming cells is not fully understood. RUNX2, OSX, OPN, OCN and BMP4 are both involved in tooth development^16, 17^. RUNX2 expression has been reported in the mesenchyme of teeth and bones^18^. Further, GATA4 has been shown to regulate osteoblast differentiation *via* RUNX2^19^. BMP4 gene was identified as a direct downstream target for GATA4^20^. However, the specific interaction of GATA4 with these transcription factors during tooth development has not been elucidated. In this study, we demonstrated that GATA4 knockdown in dental mesenchymal cells significantly reduced the expression of RUNX2, OSX, OPN, OCN and BMP4 in the dental mesenchymal cells around the root. We also assessed the expression of these odonto/osteogenic markers *in vitro* by inducing GATA4 knock-down in the SCAPs. The proliferation was found to be affected both *in vivo* and *in vitro*. Collectively, our data fits best with a model in which GATA4 promotes root development, and affects the expression of odonto/osteogenic markers to stimulate proliferation of dental mesenchymal cells around the apical area of root, thereby enabling its growth and elongation.

Guanine nucleotide binding proteins are expressed widely in cells and work as switches to transmit signals from outside to the cells^21^. G protein signaling is involved in bone development, remodeling, and disease^22^. GNAI3 is one of the members of G protein family. GNAI3^−/−^ mutant mice have abnormal bone phenotype caused by targeted deletion of GNAI3 in mice which induces fusion of ribs and vertebrae^23^.

GNAI3 has also been identified in the majority of cases of the auriculocondylar syndrome (ACS). ACS is a rare craniofacial deformity that involves neural crest derived first and second pharyngeal arch (PA) derivatives; malformations include micrognathia, temporomandibular joint and condylar anomalies^11, 12^. The hallmark features of ACS such as mandibular hypoplasia are consistent with the mouse model *Wnt1-Cre;GATA4* ^*fl/fl*^ (data not shown). Mutants exhibited widened cranial frontal sutures and mandibular retrognathism. Von Kossa, total collagen and collagen-I staining revealed a significant decrease in mineralization in the cranial neural crest-derived bone of *Wnt1-cre;GATA4* ^*fl/fl*^ mice, as compared to that in control mice. Besides, in our previous work, iTRAQ assay showed that GATA4 knock-down in the neural crest cells decreased the expression of GNAI3. Our findings in the dental mesenchymal cell of *Wnt1-Cre;GATA4* ^*fl/fl*^ mice and SCAPs are in line with our earlier findings. Collectively, GNAI3 appears to be regulated by GATA4 during the tooth root development. However, the specific mechanism of GNAI3 on tooth morphogenesis needs further exploration.

Our work substantiates the involvement of both GATA4 and GNAI3 in root development. The results therefore provide valuable insights into the etiology of short root defects and open the front for further studies on signaling molecules that promote tooth development.

## Materials and Methods

### Animals

All experiments were performed with the approval of the Ethics Committee of the Stomatological School of Nanjing Medical University. All procedures were performed according to the guidelines of the Animal Care Committee of Nanjing Medical University. Mice were obtained from the Model Animal Research Center (MARC) at the Nanjing University. To delete GATA4 specifically in the dental papilla mesenchymal cells, we crossed *Wnt1-Cre;GATA4* ^*fl/*+^ males with *GATA4* ^*fl/fl*^ females (C57/BL6).

### Micro-CT analysis

Micro-CT datasets were obtained using a micro-CT scanner (Skyscan 1176, Kontich, Belgium). Genetically modified mice were sacrificed at various time points. The skulls were dissected free of soft tissue, and fixed in 70% ethanol overnight. The slice thickness for micro-CT scans was 18 μm at 50 kV and 456 μA^24^. Images were reconstructed and analyzed by NRecon v1.6 and CTAn v1.13.8.1 software (Bruker, Germany).

### Histological analysis

*Wnt1-Cre;GATA4* ^*fl/fl*^ and *GATA4* ^*fl/fl*^ mice were harvested at embryonic day 14.5 (E14.5), 15.5 (E15.5), 17.5 (E17.5) and at postnatal days 1 and 14. Skull were carefully dissected and fixed in freshly prepared 4% paraformaldehyde (PFA). For 14-day-old mice, tissues were decalcified in 10% EDTA solution for 2–3 weeks. Decalcified tissues were dehydrated, paraffin-embedded and 5-µm thick sections prepared. Hematoxylin and eosin (HE) staining^25^ and immunohistochemical examination was performed to examine the phenotypic changes and molecular expressions^26^. For immunohistochemical examination, the tissue sections were treated with 3% H_2_O_2_ for 30 minutes to remove endogenous tissue peroxidase. Then, the sections were washed three times (5 mins each) using 0.1 M Phosphate buffered saline (PBS). The sections were treated with normal goat serum for 30 mins at 37 °C. Polyclonal rabbit anti-mouse GATA4, anti-mouse Nestin, anti-mouse RUNX2, anti-mouse OSX, anti-mouse OPN, anti-mouse OCN, anti-mouse BMP4, anti-mouse PCNA and anti-mouse GNAI3 antibodies (all obtained from Abcam, USA) were used as primary antibodies at dilutions of 1:300 for 30 min at 37 °C. The sections were washed thrice using 0.1 mol/L PBS. Finally, immune complexes were visualized using diaminobenzidine (DAB) kit, and the sections counterstained with hematoxylin^27^.

### Isolation and culture of human stem cells of dental apical papilla

Human stem cells of dental apical papilla (SCAPs) isolated from impacted third molars were acquired from patients (age: 16–20 years) treated at the Oral Surgery Department of the Jiangsu Provincial Stomatological Hospital (Nanjing, China). The Ethical Committee at the Stomatological School of Nanjing Medical University (Nanjing, China) approved all experiments; written informed consent was obtained from the patients. The apical papilla tissues were gently separated from the root apex, minced and digested in a solution containing alpha minimum essential medium (α-MEM, Gibco, Life Technologies, Grand Island, USA), 3 mg/mL collagenase type I (Sigma Aldrich Chemie, Taufkirchen, Germany) and 4 mg/mL dispase (Roche, Mannheim, Germany) for 30 min at 37 °C to obtain primary cells^9^. Single cell suspensions were obtained and cultured in α-MEM medium supplemented with 10% fetal bovine serum, 2 mmol/L glutamine, 100 U/mL penicillin and 100 mg/mL streptomycin at 37 °C in a 5% CO_2_ incubator. Culture media were changed every 3 days. The cells were passaged at a ratio of 1:2 when they reached 80–90% confluence. SCAPs at passage 2–5 were used in the subsequent experiments^28^.

### Flow cytometric analysis

SCAPs were used to evaluate stem cell surface markers on flow cytometric (FCM) analysis. Briefly, cell suspensions were collected and incubated in the dark for 1 h at 4 °C with CD29 APC, CD90 PE CY7, CD34-PE and CD45-FITC antibodies (BD Biosciences, San Jose, USA)^24^. Cells were washed twice in PBS and the expression profiles analyzed by flow cytometry following staining^29^.

### Lentiviral transfection

Recombinant lentivirus of shRNA target GATA4 (shGATA4; 5′-GAATAAATCTAAGACACCA-3′), GNAI3 (shGNAI3; 5′-CCAGGGAAUAUCAGCUCAATT-3′) and control lentivirus (shCTRL; 5′-TTCTCCGAACGTGTCACGT-3′) were purchased from GenePharma (Shanghai, China). Lentivirus to overexpress GATA4 (pcDNA-GATA4) and blank lentivirus were designed and constructed by GenePharma (Shanghai, China). SCAPs were seeded at a density of 1.5 × 10^5^ cells/well in 6-well culture plates, incubated overnight, and subsequently infected with the lentivirus (MOI = 50) at 60–70% confluence in the presence of 5 μg/mL Polybrene^30^. The medium was switched to a normal medium 24 h after transfection. Efficiency of lentiviral infection was measured by fluorescence microscopy three days later (Leica Microsystems, Ontario, Canada). Western blotting was performed to verify GATA4 knock-down.

### Wound scratch assay

SCAPs transfected with GATA4 lentivirus were seeded on to 6-well plates at a density of 1.5 × 10^5^ cells/well. When cells grew to 95–100%, a pipette tip was used to inflict a wound. Debris and floating cells were removed by washing the cells once with 1 mL of the growth medium. Wound closure was measured at different time points (12 and 24 h) using a Leica DMIRE2 microscope in phase contrast mode and Leica FW4000 software (Cambridge, UK)^31^.

### Cell counting kit-8 assay

SCAPs transfected with GATA4 lentivirus were seeded on to 96-well plates at a density of 1000 cells/well and cultured in α-MEM with 10% FBS. The cells infected with control lentivirus served as control groups. At specific time points (0 h, 24 h, 48 h, 72 h, 96 h), the cultures were supplemented with cell counting kit-8 (CCK-8) solution (Dojindo, Japan) and incubated for 1 h at 37 °C. Cell proliferation was measured at a wavelength of 450 nm by a microplate reader^32^.

### Alkaline phosphatase activity assay

SCAPs were cultured in mineralization-inducing media for 5 days, fixed with 4% paraformaldehyde, and stained with BCIP/NBT Alkaline Phosphatase Color Development Kit (Beyotime Institute of Biotechnology, Shanghai, China), according to the manufacturer’s instructions^7^. Alkaline Phosphatase (ALP) activity was measured with an ALP activity kit, according to the manufacturer’s protocol (Beyotime Institute of Biotechnology, Shanghai, China). ALP activity was determined by absorbance measured at 405 nm by an automatic microplate reader^33^.

### Alizarin red staining

Cells were cultured in mineralization-inducing medium for 2 weeks, fixed with 75% ethanol, and stained with 2% Alizarin red (Beyotime Institute of Biotechnology, Shanghai, China). Alizarin red was destained using 10% cetylpyridinium chloride (CPC) in 10 mM sodium phosphate for 30 min at room temperature for quantitative estimation of calcium mineral content. The concentration was evaluated by measuring the absorbance at 562 nm on a 96-well plate reader^34, 35^.

### Western Blotting

Details of the Western blot protocol are described elsewhere^36^. Briefly, total protein was collected using cell lysis reagent containing a protease inhibitor phenylmethanesulfonyl fluoride (PMSF). Protein samples were boiled for 5 min, loaded onto a 10% SDS-PAGE gel for separation, and transferred onto polyvinylidene fluoride (PVDF) membranes at 300 mA for one hour. After blocking with 5% bovine serum albumin (BSA) for 2 hours, the membrane was incubated overnight at 4 °C with primary antibodies against GATA4 (Abcam, USA), dentin sialophosphoprotein (DSPP, Abcam, USA), runt-related transcription factor 2 (RUNX2, Abcam, USA), osterix (OSX, Abcam, USA), osteopontin (OPN, Abcam, USA), osteocalcin (OCN, Abcam, USA), bone morphogenetic protein 4 (BMP4 Abcam, USA), GNAI3 (Abcam, USA) and GAPDH (Bioworld, China). Subsequent to this, the membranes were incubated with secondary antibodies at room temperature for 1 hour, rinsed with Tris Buffered Saline (with Tween-20) three times, and visualized by enhanced chemiluminescence. Semi-quantitative measurements were carried out using Image J software (National Institutes of Health, USA).

### Quantitative reverse transcription polymerase chain reaction for messenger RNA analysis

Total cell RNA was isolated using TRIzol reagent (Invitrogen, New York, USA) according to the manufacturer’s protocol^37^. mRNA was converted to complementary deoxyribonucleic acid (cDNA) using a PrimeScript RT reagent kit (TaKaRa Biotechnology, Dalian, China). The gene expression level was analyzed by quantitative reverse transcription PCR (qRT-PCR) using the ABI-7300 Real-Time PCR System (Applied Biosystems, CA, USA). The primers used are listed below (forward/reverse):

RUNX2 (5′-AGTTCCCAAGCATTTCATC-3′/5′-GGCAGGTAGGTGTGGTAGT-3′);

OSX (5′-CTACCCATCTGACTTTGCTC-3′/5′-CACTATTTCCCACTGCCTT-3′);

OPN (5′-CTCCAATCGTCCCTACAGTCG-3′/5′-CCAAGCTATCACCTCGGCC-3′);

OCN (5′-CACACTCCTCGCCCTATT-3′/5′-GGTCTCTTCACTACCTCGCT-3′);

BSP (5′-GCTGATGAACGCCTACTGC-3′/5′-AAACCTCGATGGTGTCGC-3′);

DSPP (5′-CCATTCCAGTTCCTCAAA-3′/5′-GCCTTCCTCTATCACCTTC-3′);

GNAI3 (5′-GGGAAGACAAATGAAAGAGAA-3′/5′-CCAACAAAGGCACTGAAC-3′);

GAPDH (5′-TGAACCATGAGAAGTATGACAACA-3′/5′-TCTTCTGGGTGGCAGTG-3′).

### Culture of neural crest cells

Embryos were extracted from gestational day E10.5 mice. Micro scissors and scalpels were used to remove decidua. Then the first branchial arch was extracted in PBS and the tissue dissociated in 0.25% trypsin-EDTA (Gibco, USA) for 30 min at 37 °C. Trypsinization was neutralized by fetal bovine serum (FBS) and cell suspensions were cultured in collagen-coated dishes in F12/DMEM (1:1, vol/vol) medium (DMEM composition: 10% FBS supplemented with penicillin, streptomycin, L-glutamate and sodium pyruvate).

### Isobaric tags for relative and absolute quantitation (ITRAQ) assay

Total proteins in each sample were extracted from the NCCs transfected with lentivirus, the supernatant was reduced and alkylated by 10 mM DL-Dithiothreitol and 55 mM iodoacetamide (IAA). After reduction and alkylation, proteins from each group were digested overnight with trypsin at 37 °C. Finally, samples were labeled with iTRAQ reagents (Sigma, USA)^38^. Samples from both the conditions were pooled and subjected to fractionation. The lentivirus used is listed below:

shGATA4 (5′-GAATAAATCTAAGACACCA-3′).

### Plasmid construction

The predicted GATA4 binding site, with the SpeI/XbaI enzyme site and the oligonucleotide sequence (F: 5′-CAGCCTCCGGACTCTAGC-3′; R: 5′-TAATACGACTCACTATAGGG-3′) was synthesized, and cloned into the SpeI/XbaI site of the pEZX-FR03 luciferase vector after reannealment. The pEZX-FR03 luciferase vector contained the GATA4 binding site was constructed at the same time.

### Dual-luciferase assay

293 T cells were cultured in a 24-well plate at a density of 1.0 × 10^5^ cells/well. At 80% confluence, the cells were cotransfected with pEZ-GATA4, pEZ-GNAI3 promoter-luciferase (400 ng/well) and plasmid pEZ-SV40 (10 ng/well) utilizing Polyetherimide (PEI) (23966-2, polysciences, Warrington, PA, USA). After 48 hours, luciferase activity was assayed using dual-luciferase reporter assay kit (Promega, Madison, WI, USA). Levels of firefly luciferase were standardized to those of Renilla^39^. All samples were measured at least 5 times.

### ChIP (chromatin immunoprecipitation) assay

ChIP assays were performed using EZ-ChIP (Millipore Corporation) according to the manufacturer’s protocol^40^. Briefly, SCAPs were cross-linked with 1% formaldehyde at 37 °C for 10 min; subsequently, the cells were lysed in SDS buffer to shear DNA by sonicate. Lysates diluted with ChIP dilution buffer were immunoprecipitated with anti-GATA4 (ab86371, Abcam) or rabbit IgG as an internal control. PCRs were performed to amplify the fragment of GNAI3 promoter using 2 μL of the extracted DNA (with or without antibody) as a template. PCR products were electrophoresed on 2% agarose gel stained with ethidium bromide and visualized under UV light.

### Statistical analysis

All experiments in this study were performed three times to test the reliability of the results and important observations are discussed in this article. Experimental values are expressed as mean ± Standard Deviation (SD). The results in the control and treatment groups were assessed by Student’s *t*-test (SPSS 19.0). *P* < 0.05 was considered statistically significant.

## References

[1] Thesleff I (2003). Epithelial-mesenchymal signalling regulating tooth morphogenesis. Journal of cell science.

[2] Luan X, Ito Y, Diekwisch TG (2006). Evolution and development of Hertwig’s epithelial root sheath. Developmental dynamics: an official publication of the American Association of Anatomists.

[3] Molkentin JD (2000). The zinc finger-containing transcription factors GATA-4, -5, and -6 ubiquitously expressed regulators of tissue-specific gene expression. The Journal of biological chemistry.

[4] Zhou P, He A, Pu WT (2012). Regulation of GATA4 transcriptional activity in cardiovascular development and disease. Current topics in developmental biology.

[5] Heineke J (2007). Cardiomyocyte GATA4 functions as a stress-responsive regulator of angiogenesis in the murine heart. The Journal of clinical investigation.

[6] Wiszniak S (2015). Neural crest cell-derived VEGF promotes embryonic jaw extension. Proceedings of the National Academy of Sciences of the United States of America.

[7] Guemes M (2014). GATA4 is essential for bone mineralization *via* ERα and TGFβ/BMP pathways. Journal of bone and mineral research: the official journal of the American Society for Bone and Mineral Research.

[8] Miranda-Carboni GA (2011). GATA4 regulates estrogen receptor-alpha-mediated osteoblast transcription. Molecular endocrinology.

[9] Sonoyama W (2008). Characterization of the apical papilla and its residing stem cells from human immature permanent teeth: a pilot study. Journal of endodontics.

[10] Kuo CT (1997). GATA4 transcription factor is required for ventral morphogenesis and heart tube formation. Genes & development.

[11] Gordon CT (2013). Mutations in endothelin 1 cause recessive auriculocondylar syndrome and dominant isolated question-mark ears. American journal of human genetics.

[12] Romanelli Tavares VL (2015). Novel variants in GNAI3 associated with auriculocondylar syndrome strengthen a common dominant negative effect. European journal of human genetics.

[13] Katsuragi Y (2013). Bcl11b transcription factor plays a role in the maintenance of the ameloblast-progenitors in mouse adult maxillary incisors. Mechanisms of development.

[14] Kim TH (2015). Osterix regulates tooth root formation in a site-specific manner. Journal of dental research.

[15] Wu LA (2016). Establishment of immortalized BMP2/4 double knock-out osteoblastic cells is essential for study of osteoblast growth, differentiation, and osteogenesis. Journal of cellular physiology.

[16] Graf D, Malik Z, Hayano S, Mishina Y (2016). Common mechanisms in development and disease: BMP signaling in craniofacial development. Cytokine & growth factor reviews.

[17] Hirata A, Sugahara T, Nakamura H (2009). Localization of runx2, osterix, and osteopontin in tooth root formation in rat molars. The journal of histochemistry and cytochemistry: official journal of the Histochemistry Society.

[18] D’Souza RN (1999). Cbfa1 is required for epithelial-mesenchymal interactions regulating tooth development in mice. Development.

[19] Song I (2014). GATA4 negatively regulates osteoblast differentiation by downregulation of Runx2. BMB reports.

[20] Nemer G, Nemer M (2003). Transcriptional activation of BMP-4 and regulation of mammalian organogenesis by GATA-4 and -6. Developmental biology.

[21] McCudden CR, Hains MD, Kimple RJ, Siderovski DP, Willard FS (2005). G-protein signaling: back to the future. Cellular and molecular life sciences.

[22] Wu M, Deng L, Zhu G, Li YPG (2010). Protein and its signaling pathway in bone development and disease. Frontiers in bioscience: a journal and virtual library.

[23] Plummer NW (2012). Development of the mammalian axial skeleton requires signaling through the Galpha(i) subfamily of heterotrimeric G proteins. Proceedings of the National Academy of Sciences of the United States of America.

[24] Guo S (2016). Class A Scavenger Receptor Exacerbates Osteoclastogenesis by an Interleukin-6-Mediated Mechanism through ERK and JNK Signaling Pathways. International journal of biological sciences.

[25] Xavier GM (2015). Activated WNT signaling in postnatal SOX2-positive dental stem cells can drive odontoma formation. Scientific reports.

[26] Ono W, Sakagami N, Nishimori S, Ono N, Kronenberg HM (2016). Parathyroid hormone receptor signalling in osterix-expressing mesenchymal progenitors is essential for tooth root formation. Nature communications.

[27] Chen W (2014). Cbfbeta deletion in mice recapitulates cleidocranial dysplasia and reveals multiple functions of Cbfbeta required for skeletal development. Proceedings of the National Academy of Sciences of the United States of America.

[28] Wan F (2015). Proliferation and osteo/odontogenic differentiation of stem cells from apical papilla regulated by Zinc fingers and homeoboxes 2: An *in vitro* study. Biochemical and biophysical research communications.

[29] Zhang W (2014). Proliferation and odontogenic differentiation of BMP2 genetransfected stem cells from human tooth apical papilla: an *in vitro* study. International journal of molecular medicine.

[30] Zhang H (2015). Canonical Wnt signaling acts synergistically on BMP9-induced osteo/odontoblastic differentiation of stem cells of dental apical papilla (SCAPs). Biomaterials.

[31] Li J (2014). Effects of canonical NF-κB signaling pathway on the proliferation and odonto/osteogenic differentiation of human stem cells from apical papilla. BioMed research international.

[32] Yu S, Zhao Y, Ma Y, Ge L (2016). Profiling the secretome of human stem cells from dental apical papilla. Stem cells and development.

[33] Li Y (2014). 17beta-estradiol promotes the odonto/osteogenic differentiation of stem cells from apical papilla *via* mitogen-activated protein kinase pathway. Stem cell research & therapy.

[34] Qu B (2014). Distal-less homeobox 2 promotes the osteogenic differentiation potential of stem cells from apical papilla. Cell and tissue research.

[35] Fan Z (2009). BCOR regulates mesenchymal stem cell function by epigenetic mechanisms. Nature cell biology.

[36] Cao Z (2015). Osterix controls cementoblast differentiation through downregulation of Wnt-signaling *via* enhancing DKK1 expression. International journal of biological sciences.

[37] Aioub M (2007). Msx2^−/−^ transgenic mice develop compound amelogenesis imperfecta, dentinogenesis imperfecta and periodental osteopetrosis. Bone.

[38] O’Brien RN, Shen Z, Tachikawa K, Lee PA, Briggs SP (2010). Quantitative proteome analysis of pluripotent cells by iTRAQ mass tagging reveals post-transcriptional regulation of proteins required for ES cell self-renewal. Molecular & cellular proteomics: MCP.

[39] Yang F (2014). NEXN inhibits GATA4 and leads to atrial septal defects in mice and humans. Cardiovascular research.

[40] Kadariya Y (2005). Regulation of human methylthioadenosine phosphorylase gene by the CBF (CCAAT binding factor)/NF-Y (nuclear factor-Y). The Biochemical journal.

